# Genetic and metal analyses of fragmented populations of *Betula papyrifera* (Marsh) in a mining reclaimed region: identification of population–diagnostic molecular marker

**DOI:** 10.1002/ece3.1195

**Published:** 2014-08-19

**Authors:** Gabriel Theriault, Kabwe K Nkongolo, Paul Michael

**Affiliations:** 1 Department of Biology, Laurentian University935 Ramsey Lake Road, Sudbury, Ontario, Canada, P3E-2C6; 2 Biomolecular Science Program, Laurentian University935 Ramsey Lake Road, Sudbury, Ontario, Canada, P3E-2C6

**Keywords:** *Betula papyrifera*, gene flow, metal accumulation, Northern Ontario (Canada), population fragmentation

## Abstract

White birch (*Betula papyrifera*) is an open pollinate species that is, dominant in the Northern Ontario after land reclamation. In fact, this species represents 65% of all trees in the region. We hypothesized that the exchange of genetic information between fragmented populations by range-wide paternal introgression is possible in wind-pollinated species such as *B. papyrifera*. On the other hand, the effects of heavy metal contamination from the mining activities on plant growth and population dynamics are well documented. The main objectives of this study were (1) to assess the level of genetic variation, gene flow, and population sustainability of *B. papyrifera* after land reclamation; and (2) to determine the level of phytoavailable metals in soil and their accumulation in trees. We found that *B. papyrifera* is a Ni and Zn accumulator with a translocation factor of 6.4 and 81, respectively, and an indicator of Cu and Pb. The level of polymorphic loci, Shannon index, Nei's genetic diversity, observed number of alleles, and gene flow were determined for the fragmented populations within the targeted region. The percent of polymorphic loci ranged from 28% to 56%; the gene flow was also low with a value of 0.89, and the population differentiation was very high with a value of 0.36. Two population–diagnostic ISSR markers were identified. They were cloned, sequenced, and converted to SCAR markers. Overall, the fragmented populations of *B. papyrifera* in Northern Ontario are genetically sustainable based on the moderate level of intrapopulation variability.

## Introduction

**Figure 1:**
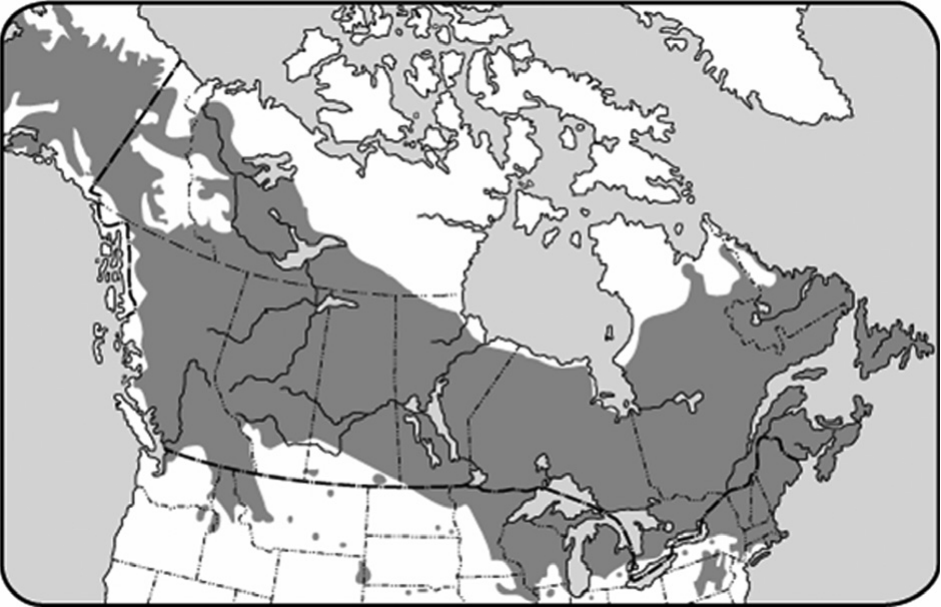
Distribution of white birch (*Betula papyrifera*) in North America according to National Resources Canada.

The genus *Betula* belonging to the *Betulaceae* family, consists of roughly 60 species of hardwood. *Betula papyrifera* (white birch), is a major component of the boreal forest of North America. It is found in every Province of Canada, except Nunavut, as well as the northern United States (Fig.1). It is considered a pioneer species; therefore, it is the first to colonize open areas after disasters such as forest fires and deforestation. Little is known about the effects of environmental pollution on white birch because of its low economic value. Studies have shown that this species is sensitive to pH changes and heavy metal contamination (McCall et al. 1995). However, others believe that long-term exposure to heavy metal contamination has lead to resistant *B. papyrifera* populations in the Northern Ontario (Kirkey et al. 2012).

Since 1883, the Greater Sudbury region located in Northern Ontario has been well known for its rich deposits in nickel, copper, and iron ores. The discovery of the deposits led to the rapid boom of the mining industry and the establishment of many mining companies within the region. For this reason, it is also known as one of the Canada's most ecologically disturbed regions. Large quantities of sulfur dioxide and heavy metal particles were released from smelters and roast yards (Freedman and Hutchinson 1980). This caused heavy metal contamination and acidification of soils (Freedman and Hutchinson 1980; Winterhalder 1995; Gratton et al. 2000). In combination with deforestation, important soil nutrients were washed away by erosion (Lautenbach 1987). Sites surrounding smelters were completely barren of vegetation with only a few tree species remaining, with white birch being one of the main ones (Amiro and Courtin 1981). Restoration efforts began in 1973 to reclaim the disturbed lands through seedling planting, fertilization, and liming. This, along with emission reductions, has helped restore vegetation and nutrient-rich soil horizons (Theriault et al. 2013).

White birch (*B. papyrifera*) is an open pollinate species that is, dominant in the Northern Ontario after land reclamation. In fact, this species represents more than 50% of all trees in the region. Because of the long-distance pollen dispersal, gene flow among the fragmented populations is expected to be high. We hypothesized that the exchange of genetic information between fragmented populations by range-wide paternal introgression is possible in wind-pollinated species such as *B. papyrifera* and that the population differentiation will be low.

The main objectives of this study were (1) to assess the level of genetic variation, gene flow, and population sustainability of *B. papyrifera* (white birch) after land reclamation; and (2) to determine the level of phytoavailable metal in soil and accumulation in *B. papyrifera*.

## Materials and Methods

### Diversity assessment

The distribution and percentage of each plant species was determined for every site. Three quadrants with a radius of 5 m were randomly selected across the sites. They were 1 m apart. Tree species richness in each quadrant of each tree species was calculated based on direct counting. Individuals with trunks larger than 10 cm were counted as trees and those smaller as saplings.

### Metal analysis

**Figure 2:**
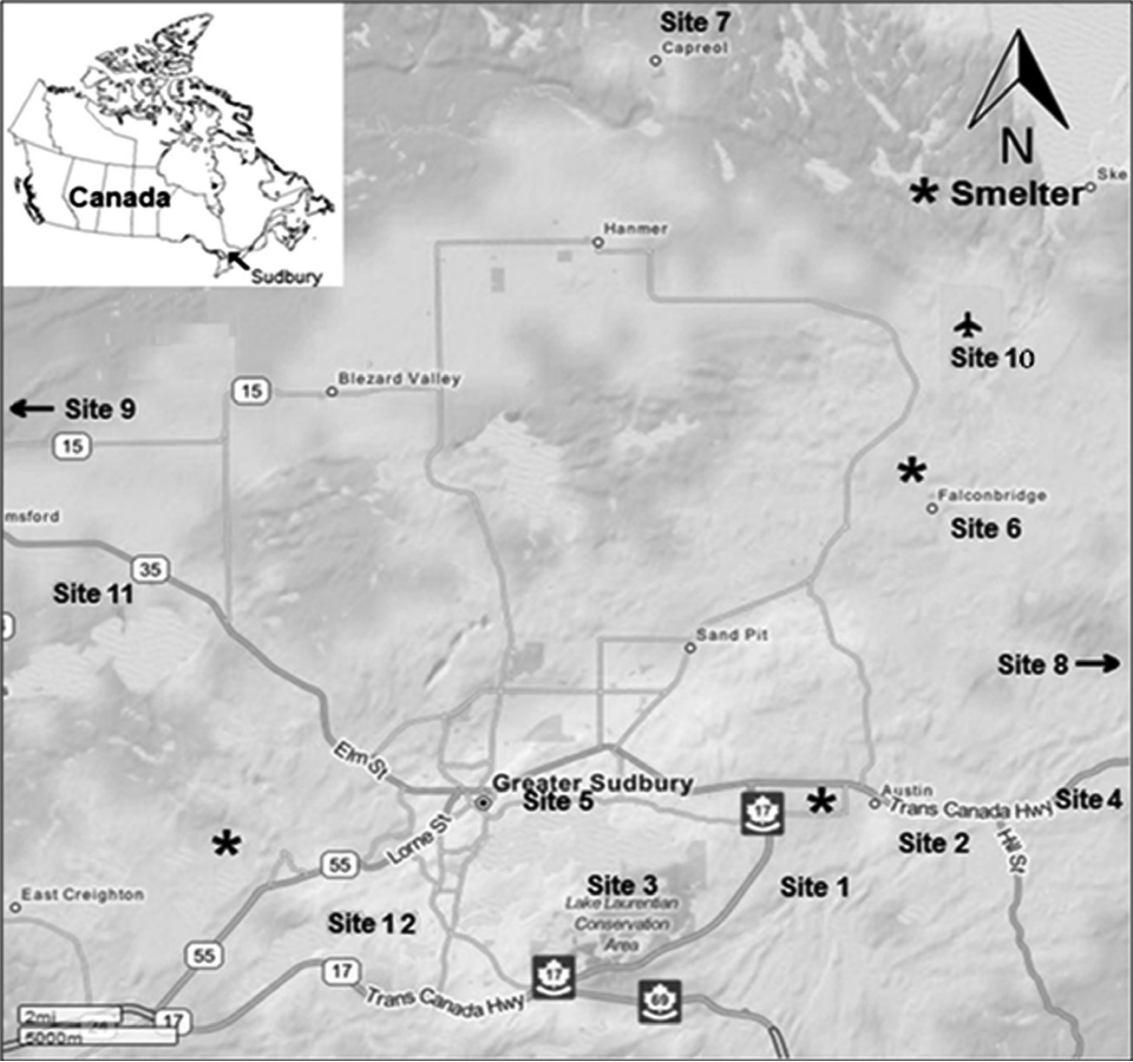
Location of white birch sampling sites in Northern Ontario within the Greater Sudbury region. Site 1: Daisy Lake; Site 2: Wahnapitae Hydro-Dam; Site 3: Laurentian; Site 4: Kukagami; Site 5: Kingsway; Site 6: Falconbridge; Site 7: Capreol (reference); Site 8: St. Charles (reference); Site 9: Onaping Falls (reference); Site 10: Airport; Site 11: Azilda; Site 12: Kelly Lake.

Soil samples were collected across the twelve sites (Fig.2). These sites were selected based on varying distances from the smelters. Only the top soil layer was analyzed for this study. Total and phytoavailable metals were determined as described by Abedin et al. (2012) and Nkongolo et al. (2013). To measure total metals, soil or leaf samples were first digested in aqua regia. Roughly 0.5–0.05 g of the sample were digested with 5 mL of concentrated HNO_3_ and HCL using a MARS 5 microwave oven. The supernatant was transferred and brought up to 50 mL with deionized water. Metal levels were measured using an inductively coupled plasma optical emission spectrometry (ICP-OES), inductively coupled plasma mass spectrometry (ICP-MS), and hydride generation atomic emission spectrometry (HG-AAS).

Bioavailable (phyto) metals were measured by adding 5 g of soil with 20 mL of 0.01 mol/L LiNO_3_ to 50-mL centrifuge tubes. Tubes were placed in a shaker under ambient lighting conditions for 24 h at 20°C (Abedin et al. 2012). The pH (LiNO_3_) of the suspension was measured prior to centrifugation at 1100 × g for 20 min, with filtration of the supernatant through a 0.45-*μ*m filter into a 20-mL polyethylene tube and made to volume with deionized water. The filtrate was preserved at approximately 3°C for chemical analysis by ICP-MS. The quality control program completed in an ISO 17025 accredited facility (Elliot Lake Research Field Station of Laurentian University) included analysis of duplicates, internal reference materials (IRMs), procedural and calibration blanks, with continuous calibration verification, and use of internal standards (Sc, Y, Bi) to correct for any mass bias. All concentrations were calculated in mass/mass dry soil basis.

### Statistical analysis

The metal data were analyzed using SPSS 20 IBM, New York, USA, for Windows, with all data being normalized. Variance ratio test was performed with an assumption of data normality in the underlying population distributions of the data. ANOVA, followed by Tukey's HSD multiple comparison analysis, was performed to determine significant differences (*P* ≥ 0.05) among the sites. The translocation factors (TF) were determined according to the equations described by Singh and Agrawal (2008). It is estimated by the ratio of metal concentration in leaves over bioavailable metal content in soil.

### DNA extraction

White birch leaves were collected from twelve different locations across the Greater Sudbury region (Fig.2). Locations were selected based on soil contamination, previous soil treatments, and wind patterns. Up to 40 white birch trees were sampled from each population. Green leaves collected and flash frozen (duplicates) using liquid nitrogen and stored at −20°C.

Genomic DNA was extracted from each sample using a 2× CTAB buffer method, with slight modifications (Nkongolo 1999). The stock 2× CTAB buffer (2% cetyltrimethylammonium bromide, 100 mmol/L Tris-HCl [pH 8.0], 1.4 mol/L NaCl, and 20 mmol/L EDTA [pH 8.0]) was preheated each morning to 60°C in a water bath until the salt was dissolved. White birch leaves contain a lot of polyphenols and carbohydrates; therefore, a working stock was prepared everyday by adding 1% polyvinylpyrrolidone (PVP) and 0.2% *β*-mercaptoethanol to 2× CTAB buffer. Roughly 20 mL of CTAB buffer was transferred into eight 50-mL centrifuge tubes and heated in a water bath at 60°C.

Leaves were ground using liquid nitrogen until a fine powder was obtained. The powder was then transferred into the centrifuge tubes containing the buffer. The tubes were left in the water bath at 60°C for 45 min and inverted each 10 min. An equal volume of chloroform/octanol mix (24:1) was added into the tubes. The tubes were then vigorously mixed by inversion for 5 min and centrifuged (8900 × g, 15 min, 25°C). The aqueous phase (top) was transferred to a new set of tubes and washed twice more with equal volumes of 24:1 chloroform/octanol (12,500 rpm, 5 min, 25°C). An equal amount of isopropanol was added to the tubes and gently inverted to allow the DNA to precipitate. The tubes were then stored overnight at −20°C. The next day, the tubes were centrifuged (5110 × g, 5 min, 4°C). Afterwards, they were decanted and the DNA pellet was washed with 5 mL of 70% ethanol. Once completely dry, the pellet was dissolved in TE buffer (1:10 EDTA/Tris-HCl), transferred into 1.5-mL properly labelled microcentrifuge tubes, and stored at −20°C until further use.

### ISSR analysis

A total of 18 different ISSR primers synthesized by Invitrogen were used to screen a few samples from each white birch population. The DNA amplification was carried out in 0.5-mL tubes. Each reaction contained 1.5 mmol/L MgSO_4_, 1× PCR buffer, 0.5 ng/*μ*L of primer, 0.5 ng/*μ*L of dNTP stock (0.125 ng/*μ*L dATP, 0.125 ng/*μ*L dTTP, 0.125 ng/*μ*L dCTP, and 0.125 ng/*μ*L dGTP), 0.8 ng/*μ*L of DNA, 0.8 units/*μ*L of Taq polymerase (Bio Basic Inc.), and autoclaved distilled water to a final volume of 25 *μ*L. Blanks were adjusted with autoclaved distilled water instead of DNA. A drop of mineral oil was added to each tube before initiating the PCR to minimize evaporation. The PCR program consisted of an initial denaturation of 5 min at 95°C followed by a 2-min incubation at 85°C at which point the polymerase was added; 42 cycles of 1 min at 95°C, 2 min 55°C, and 1 min 72°C; a final incubation of 7 min at 72°C followed by a cooling period until 4°C.

Loading buffer was added to the PCR product ran on 2% agarose gels (3.16 V/cm). Seven primers that showed strong and reproducible amplification pattern were selected for the amplification of all the genomic DNA. The gels were documented, and bands were scored by their presence or absence using a 1 and 0 system.

Data were analyzed using the computer-based software, POPGENE 32 and Free Tree program. Popgene 32 is able to calculate multiple genetic parameters when using dominant or codominant marker systems. The following parameters were estimated, including Nei's gene diversity (*h*), Shannon's information index (*I*), and the percent of polymorphic loci per primer. The distance matrix was generated using the Jaccard's similarity coefficient analysis with the Free Tree program.

## Results

### Tree species abundance

**Figure 3:**
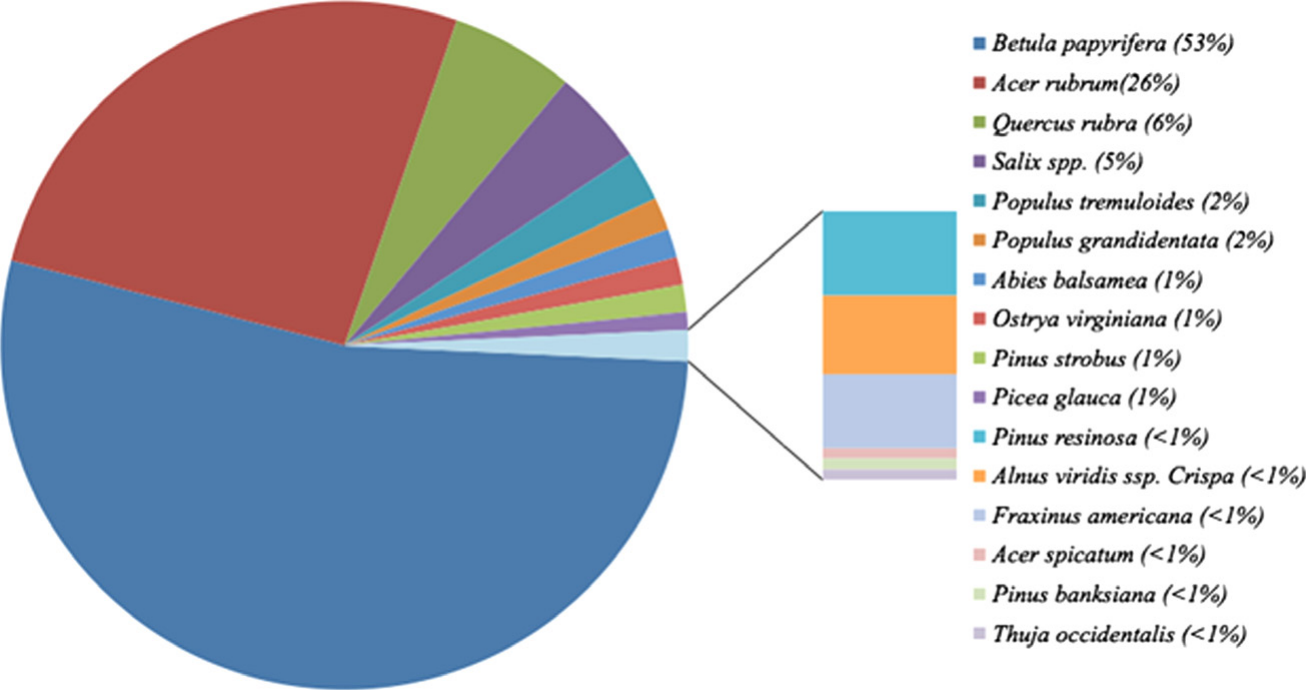
Distribution of tree and shrub species across 12 sites from the Greater Sudbury region. Sites: Capreol, Onaping Falls, St. Charles, Daisy Lake, Wahnapitae Hydro-Dam, Laurentian, Kingsway, Falconbridge, Kukagami, Azilda, Airport, and Kelly Lake.

The number of trees and shrubs was measured for each of the 12 sites. The data were pooled together to give a general idea of the distribution of the different species found in the sites (Fig.3). The fragmented plant populations in the targeted Northern Ontario region were mainly composed of hardwoods (more than 90%), with white birch (*B. papyrifera*) and red maple (*Acer rubrum*) being the predominant species. Conifers represent only a small percentage (<5%) of tree populations. Overall, *B. papyrifera* represents 53% of all trees and shrub species in the targeted region.

### Metal analysis

Detailed data on total and bioavailable heavy metals for the five elements of interest are presented in Table1. As expected, total Cu, Ni, Fe, Pb, and Zn were significantly higher in sites located around the smelters compared with reference sites. Only a very small amount of total metal was bioavailable (Table1).

**Table 1:** Total and bioavailable concentration of heavy metals in contaminated and reference soils from the Greater Sudbury region.

Metal (mg/kg)	Cu	Ni	Fe	Pb	Zn
Total					
Contaminated	991 (±242)	1192 (±336)	29200 (±3453)	736 (±141)	82 (±13)
Reference	137.67 (±25.21)	247 (±57.98)	14367 (±1625.15)	76.07 (±10.93)	81.03 (±10.75)
Bioavailable					
Contaminated	7.53 (±2.44)	4.89 (±1.57)	74.92 (±22.00)	0.23 (±0.07)	0.97 (±0.26)
Reference	0.93 (±0.61)	2.69 (±1.62)	27.21 (±11.34)	0.14 (±0.14)	2.37 (±1.09)

Contaminated sites include Daisy Lake, Wahnapitae Hydro-Dam, Laurentian, Kingsway, Falconbridge, Kukagami, Azilda, Airport, and Kelly Lake. Reference sites include St Charles, Capreol, and Onaping Falls. Standard error is shown in parenthesis.

**Figure 4:**
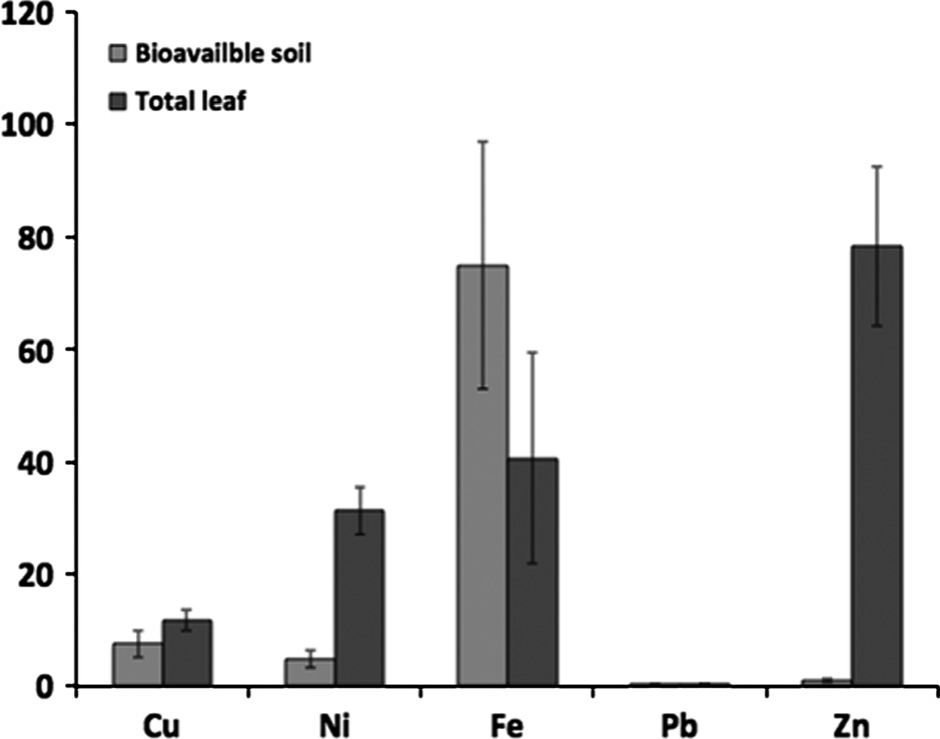
Bioavailable soil and total leaf concentration of Cu, Ni, ad Zn from contaminated sites (Daisy Lake, Wahnapitae Hydro- Dam, Laurentian, Kingsway, Falconbridge, Kukagami, Azilda, Airport, and Kelly Lake). Significant differences were found among sites (*t* ≤ 0.05).

There were significantly higher levels of Zn and Ni in leaves compared with the bioavailable amount in soil. In fact, the translocation factors were 81 and 6.4 for Zn and Ni, respectively (Fig.4). These values were 0.5, 1.6, and 1.9 for Fe, Cu, and Pb, respectively.

### ISSR analysis

Genetic diversity was measured across 12 white birch populations. The selected ISSR primers are described in Table2. The observed number of alleles (Na), the effective number of alleles (Ne), Nei's gene diversity (*h*), Shannon's information index (*I*), and the percentage of polymorphic loci (%) were also calculated (Table3).

**Table 2:** Sequence of selected ISSR primers.

Primer	Sequence (5′–3′)	G/C content (%)	Fragment number	Fragment size (bp)	Polymorphism (%)
ISSR 5	(ACG)_5_AC	65	40	280–1700	48.18
ISSR 10	(CTT) _5_(CCT) _6_CT	51	62	170–2000	33.58
ISSR 17899A	(CA) _5_AG	35	49	170–1863	55.84
UBC 825	(AC) _8_T	47	48	150–1751	48.30
UBC 827	(AC) _8_G	53	44	220–1860	56.00
UBC 841	GAAG(GA) _6_YC	56	38	200–1920	46.89
ECHT 5	(AGAC) _2_GC	60	53	145–2000	47.17

Y stands for G or C.

**Table 3:** Genetic parameters generated from white birch ISSR data using Popgene32.

Population	Na	Ne	*h*	*I*	P (%)
Metal-Contaminated					
Daisy Lake	1.3234	1.1474	0.0885	0.1369	32.34
Dam	1.4431	1.2211	0.1316	0.2016	44.31
Kingsway	1.2844	1.1311	0.0802	0.1245	28.44
Laurentian	1.5000	1.2059	0.1288	0.2035	50.00
Kukagami	1.5240	1.1981	0.1247	0.1986	52.40
Falconbridge	1.5599	1.2124	0.1345	0.2150	55.99
Airport	1.4012	1.1742	0.1066	0.1665	40.12
Azilda	1.5419	1.1982	0.1254	0.2005	54.19
References					
Capreol	1.3473	1.1374	0.0851	0.1349	34.73
St Charles	1.4551	1.1942	0.1175	0.1836	45.51
Onaping Falls	1.4491	1.1898	0.1168	0.1831	44.91

Primers are as follows UBC 825, 17889A, UBC 827, ISSR 10, ISSR 5, UBC 841, and ECHT 5. Genetic parameters are as follows observed number of alleles (Na), effective number of alleles (Ne), Nei's gene diversity (*h*), Shannon's information index (*I*), and percent of polymorphic loci (P).

The number of observed alleles (Na) ranged from 1.28 (Kingsway) to 1.56 (Falconbridge) with a mean of 1.44. The effective number of alleles (Ne) ranged from 1.13 (Kingsway) to 1.221 (Dam) with a mean of 1.18. Nei's gene diversity index (*h*) ranged from 0.08 (Kingsway) to 0.13 (Falconbridge) with a mean of 0.11. Shannon's information index (*I*) ranged from 0.13 (Kingsway) to 0.22 (Falconbridge) with a mean of 0.18. The percent of polymorphic loci (%) ranged from 28.4 (Kingsway) to 56.0 (Falconbridge) with a mean of 43.9. The gene flow was low across populations with a value of 0.89, and the population differentiation (Gst) was high with a value of 0.36. No significant difference was found between the contaminated and reference sites for all the genetic parameters estimated (Table3).

The distance matrix was generated using Jaccard's similarity coefficient (Table4). Overall, the genetic distances were high with values ranging from 0.31 (Kingsway – Wahnapitae Hydro-Dam) to 0.71 (Kingsway – St. Charles) with a mean of 0.59. There were no associations between genetic and geographic distances.

**Table 4:** Distance matrix of white birch populations from the Greater Sudbury region.

	Daisy Lake	Dam	Kingsway	Capreol	Laurentian	Kukagami	Falconbridge	St Charles	Onaping Falls	Airport	Azilda
Daisy Lake		0.6019	0.3098	0.3441	0.6383	0.6387	0.6468	0.6824	0.6303	0.6482	0.6118
Dam			0.5858	0.5952	0.5256	0.5021	0.5327	0.6750	0.5940	0.7016	0.6129
Kingsway				0.3422	0.6313	0.6255	0.6919	0.7142	0.6089	0.6615	0.6232
Capreol					0.6255	0.6379	0.6625	0.6744	0.6232	0.6536	0.6359
Laurentian						0.3805	0.4309	0.6270	0.6048	0.6302	0.6428
Kukagami							0.4008	0.6106	0.5943	0.6363	0.5686
Falconbridge								0.6299	0.6192	0.6653	0.6044
St Charles									0.5433	0.5962	0.5907
Onaping Falls										0.4951	0.5064
Airport											0.4597
Azilda											

Primers are as follows UBC 825, 17889A, UBC 827, ISSR 10, ISSR 5, UBC 841, and ECHT 5. The distance matrix was generated using Jaccard's index (Free Tree program).

### Population diagnostic molecular markers

**Figure 5:**
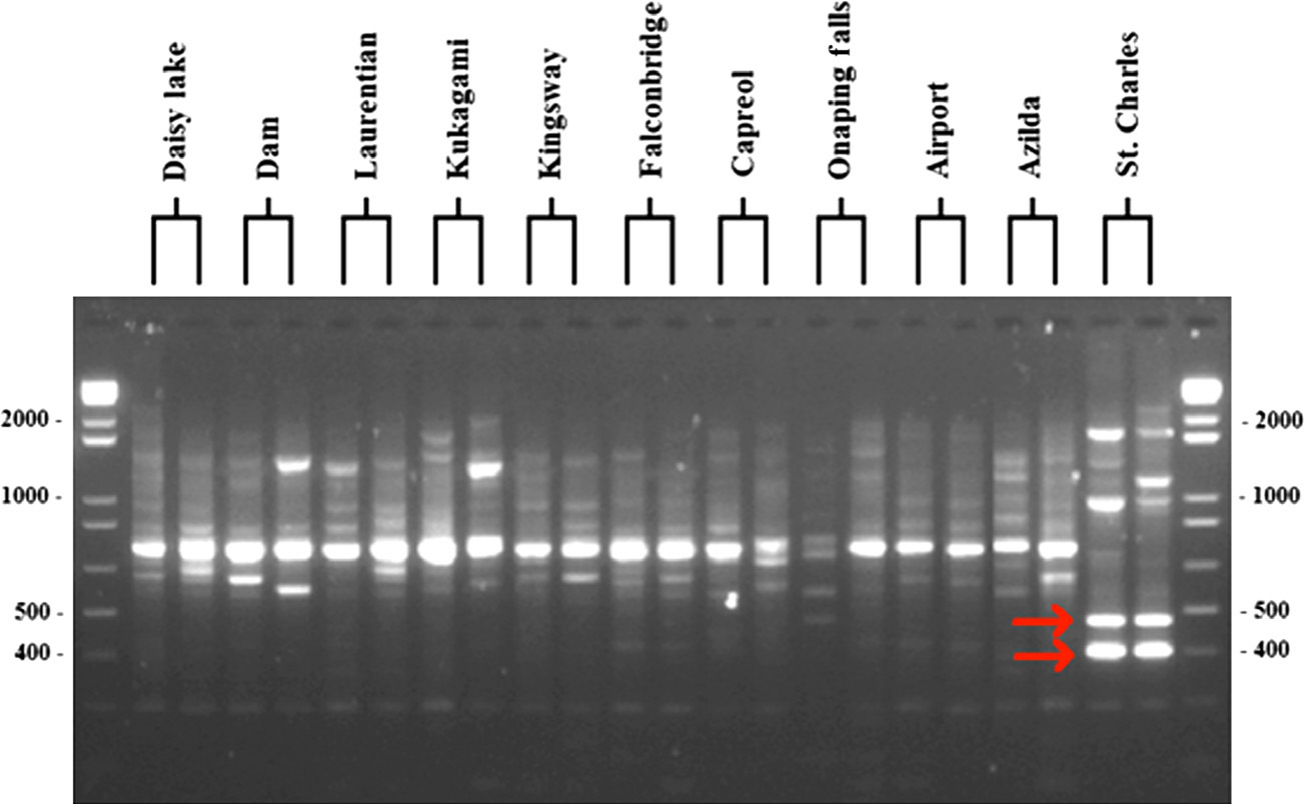
ISSR profile of 11 sites using primer ECHT 5. Population–diagnostic bands are marked by arrows.

**Figure 6:**
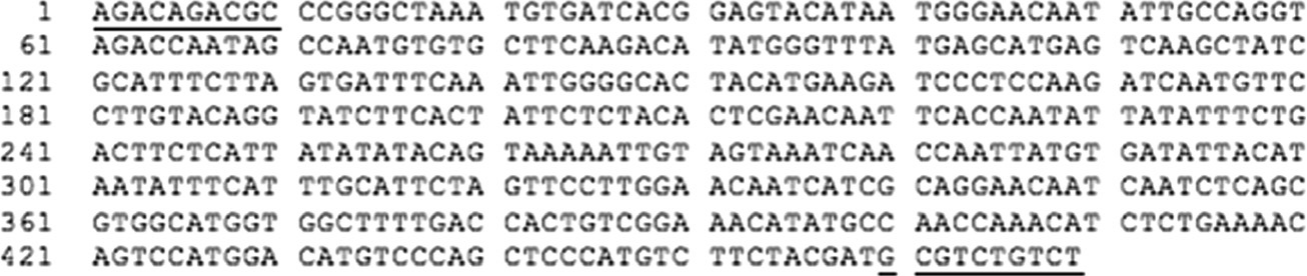
Consensus sequence of a population–diagnostic ISSR marker (469 bp) from *Betula papyrifera* using primer Echt 5 (St. Charles). ISSR primers are underlined.

**Figure 7:**
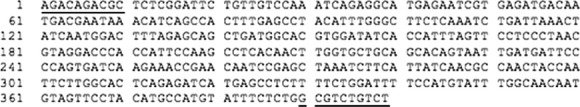
Consensus sequence of a population–diagnostic ISSR marker (399 bp) from *Betula papyrifera* using primer Echt 5 (St. Charles). ISSR primers are underlined.

Two population diagnostic ISSR markers were identified in samples from the St. Charles site (Fig.5). These diagnostic bands of 469 and 399 base pairs were extracted, cloned, and sequenced. Their consensus sequences are described in Figures6, 7. They have been registered in the GenBank under the Accession Numbers KM216986 (399 bp) and KM216987 (469 bp).

## Discussion

### Metal analysis

White birch is one of the most important tree species when it comes to the rehabilitation of disturbed regions. It is important to understand the long-term effects of heavy metal contamination on the sustainability of species populations. The number of studies analyzing the long-term effects of heavy metal contamination on the sustainability of hardwoods from Northern Ontario is very small (Theriault et al. 2013; Tran et al. 2014).

In the present study, the level of heavy metals was measured only in the organic-rich horizon (LFH) because previous studies have shown that this is where most of the airborne metal particulates reside (Nkongolo et al. 2008; Narendrula et al. 2012, 2013; Theriault et al. 2013; Tran et al. 2014). The metal data are consistent with previous studies where the bio-available form of metals was found to be significantly lower than the total amount (Theriault et al. 2013; Tran et al. 2014). This indicates that only a small fraction of heavy metals is readily available to plants; thus, toxicity should be much lower than previously thought.

The translocation factor was calculated using the bioavailable levels of heavy metals because total levels do not represent forms that are readily available to plants (Tran et al. 2014). Results show that white birch accumulates Ni and Zn in their leaves (Tran et al. 2014). No significant accumulation of Cu, Fe, and Pb in leaves was detected. This is consistent with other studies, where white birch growing in metal contaminated areas accumulated higher concentrations of heavy metals in their leaves (Dudka et al. 1996; Freedman and Hutchinson 1980). Freedman and Hutchinson (1980) found that leaves from white birch growing in close proximity to smelters had Ni and Cu concentrations of up to 170 mg/kg and 80 mg/kg, respectively. Our findings show that since then, emission control and the land reclamation program have helped lower accumulation in white birch leaves.

*Betula papyrifera* (white birch) might therefore play a role in the phytoextraction of Zn and Ni. It can be considered as indicator of Cu and Pb because the amount in leaves was similar to the bioavailable content in soil. As it represents up to 53% of all tree species in the targeted sites, it can contribute to the phytostabilization of restored forests, because it is able to grow and survive on heavily contaminated soil.

### Molecular analysis

Genetic variation is one of the key characteristics of forest sustainability. If genetic variation is low, the overall gene pool of a population will be small and could result in its extermination or a population bottleneck if a stress occurs (Nei et al. 1975). In this study, the genetic variation in 12 white birch populations was assessed using ISSR. This technique possesses many advantages compared with earlier molecular marker systems such as SSR and RFLP. With its high throughput and lack of need for genetic information, 220 samples were screened with seven ISSR primers.

Dominant markers such as ISSR provide a cost-effective approach of characterizing genetic parameters. However, the uncertainty about the underlying genotypes presents a problem for statistical analysis. The current version of POPGENE (version 32 University of Alberta, Alberta, Canada) used in the present study is designed specifically for the analysis of codominant and dominant markers using haploid and diploid data.

The percent of polymorphic loci ranged from 28.44 to 56.0. This low to moderate genetic variation is smaller than the polymorphism observed in red oak, black spruce, and jack pine populations growing in the same region (Dobrzeniecka et al. 2011; Vandeligt et al. 2011; Tran et al. 2014). The means of Nei's gene diversity index (*h*) and Shannon's information index (*I*) were low (0.113 and 0.177) indicating a low allelic frequency and uneven distribution. In the present study, the two generations were compared for genetic variation, and no significant differences in polymorphism were observed between the parental and the offspring generation. Likewise, no significant difference in genetic variation was found between white birch growing in heavy metal contaminated and reference sites. This is consistent with other studies performed on other tree species (Dobrzeniecka et al. 2011; Vandeligt et al. 2011; Tran et al. 2014). These results suggest that the current level of heavy metals and the limited number of generations are not sufficient to induce detectable selective effects on the targeted populations based on the neutral molecular markers used.

The gene flow among the fragmented populations was low despite the long-distance pollen dispersal of white birch. The exchange of genetic information between fragmented populations of this wind–pollinated species by range-wide paternal introgression appears to be limited. The genetic variation within populations was smaller than other hardwood species growing in the same areas such as red oak and red maples (Kalubi et al. 2013; Tran et al. 2014). There was no evidence of population isolation, but in general, high values for interpopulation differentiation and low gene flow suggest a risk of population divergence overtime. The values of Nm of 0.88 and Gst of 0.36 may lead to the predominance of genetic drift and low genetic variation. It has been suggested that a value of Gst lying in the range 0–0.05 indicates little genetic differentiation; a value between 0.05 and 0.15, moderate differentiation; a value between 0.15 and 0.25, great differentiation; and values above 0.25, very great genetic differentiation (Wright 1978; Hartl and Clark 1997; Balloux and Lugon-Moulin 2002). Taken together, the potentially limited capacity for dispersal among fragmented populations via both pollen and seeds may explain the high level of genetic differentiation found in white birch populations from Northern Ontario that were analyzed in the present study. This was confirmed by the data related to populations relatedness. In fact, 85% of genetic distance values were above 0.50 with an average of 0.60, suggesting that the populations analyzed were not genetically closely related.

It is, however, well established that the bulk of pollen is deposited near the source plant (Tampieri et al. 1977). However, birches as well as the other species (*Alnus, Carpinus, Corylus, Ostrya, Fagus, Quercus, Castanea*) belonging to the order Fagales are wind-pollinated trees, generating vast amounts of pollen to ensure a sufficient level of fertilization of female flowers over receptor regions. Their pollen grains are quite small and light to facilitate the transportation of a substantial fraction (up to 1% for some plumes) of the released material over thousands of kilometers if weather conditions are suitable (Sofiev et al. 2006). The limited gene flow is likely caused by local conditions rather than species characteristics. It should be pointed out that the site selection was based on different wind directions. Therefore, any effect of wind direction on introgression of alleles between populations would have been detected.

The fact that only two population diagnostic markers were identified in one reference sites suggests that the level of gene flow, although small, remains adequate to prevent population divergence. It should be pointed out that gene flow is an extremely complex phenomenon that can occur in many ways and that can fluctuate over time. The lack of a generally applicable direct method for measuring gene flow hinders its evaluation as a potential cohesive force among conspecific populations (Larson et al. 1984). But, indirect methods used in several studies based upon the distribution of allelic frequencies permit the evaluation of gene flow parameters. The values on Nm described in the present study should be taken as general estimates of “effective gene flow”.

The two population–diagnostic bands that were identified were sequenced and transformed to SCAR markers. The primers designed that flanked SCAR sequence amplified genomic DNA from all the populations, suggesting that the sequences were not truly population specific. This is an indication that the sequences are present in low copy number in all the populations, but in a higher frequency in the original population where it was identified with ISSR primers.

There exist, however, other reasons that could explain why diagnostic marker once converted to a SCAR marker is not specific. The appearance of a band in one population or species and its absence in another could be the result of competition among DNA fragments during amplification (Nkongolo 2003). Amplified products that are complementary to each other are stabilized by internal base pairing that could prevent amplification by outcompeting the binding of random primers (McGrath et al. 1998); this is the most serious problem that leads to the incorrect interpretation of results. The formation of secondary structures including hairpin by DNA fragments can also interfere with the final application outcome. As the SCAR system is a more sensitive technique, the above problems will not interfere with the amplification of sequences even if they are present in a low copy number.

## Conclusion

White birch represents more than 50% of the tree species in the targeted region following soil restoration. The total heavy metal levels were found to be high, but the availability of these metals was much lower. Compared with previous data, heavy metal levels are decreasing, possibly due to leaching and the phytoextraction by surrounding vegetation. White birch is accumulating zinc and nickel in their leaves and is indicator of Cu and Pb. The levels of genetic variation were low to moderate. Contrary to our hypothesis, the gene flow among the fragmented populations was low and population differentiation was relatively high in the reclaimed mining region. Populations within the targeted region were distantly related, and diagnostic markers were identified only for one of the 12 populations. No association between the levels of genetic variation in white birch populations and heavy metal content in the soil was established. The genetic analysis revealed that white birch populations from the Greater Sudbury region are genetically sustainable.
